# Subcutaneous emphysema of the scrotum (pneumoscrotum) due to traumatic pneumothorax: a case report

**DOI:** 10.1186/1757-1626-1-293

**Published:** 2008-11-01

**Authors:** Vasileios Simaioforidis, Stylianos Kontos, Ioannis Fokitis, Georgios Lefakis, Sotirios Koritsiadis

**Affiliations:** 1 Department of Urology, General Hospital of Nikea, 3 D. Mantouvalou St., Nikea, 18454, Piraeus, Greece

## Abstract

**Introduction:**

Subcutaneous emphysema of the scrotum due to traumatic pneumothorax is a rare medical situation and only a few cases are reported in the literature.

**Case report:**

We present the case of a 22 year old man who was admitted to the emergency department after a motorcycle accident having a painless crepitant scrotum and chest excoriations. Further evaluation revealed subcutaneous emphysema of the scrotum caused by left pneumothorax.

**Conclusion:**

In conclusion, subcutaneous emphysema of the scrotum (or pneumoscrotum) due to traumatic pneumothorax is not an urgent condition and assessment should be supportive with intervention directed at the etiology, e.g. the pneumothorax.

## Background

Subcutaneous emphysema of the scrotum (or pneumoscrotum) is a rare condition and receives little or no discussion in standard texts of urology. This paper presents a case of pneumoscrotum due to traumatic pneumothorax and discusses its clinical course, findings and treatment strategy with a review of the relevant literature.

## Case presentation

**Figure 1:**
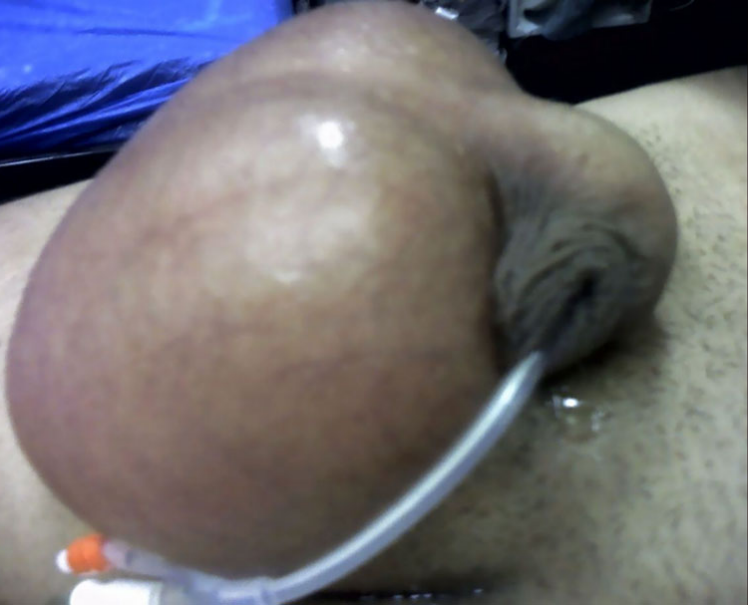
Oedematous scrotum (profile).

A 22 year old man was admitted to the emergency department following a motorcycle accident. He had chest excoriations and a painless crepitant scrotum (Fig 1, 2.). Palpation of the scrotum was characteristic and gave the impression of palpating "snow". Testicles could not be palpated. During clinical examination, patient complained of incipient dyspnea. Auscultation of the lungs placed the suspicion of pneumothorax. The CT imaging study demonstrated a left pneumothorax (Fig. 3) causing a subcutaneous emphysema that was expanded in the scrotum (Fig. 4). A left haemothorax and three rib fractures were also diagnosed. No lesions of the genitalia were diagnosed. A chest drainage tube was inserted and further assessment was supportive in collaboration with the thoracosurgery clinic. Patient was dismissed eight days after the accident. Pneumothorax was partially absorbed and further control was arranged as an outpatient. By one month, no air was found in the ultrasound imaging of the scrotum.

**Figure 2:**
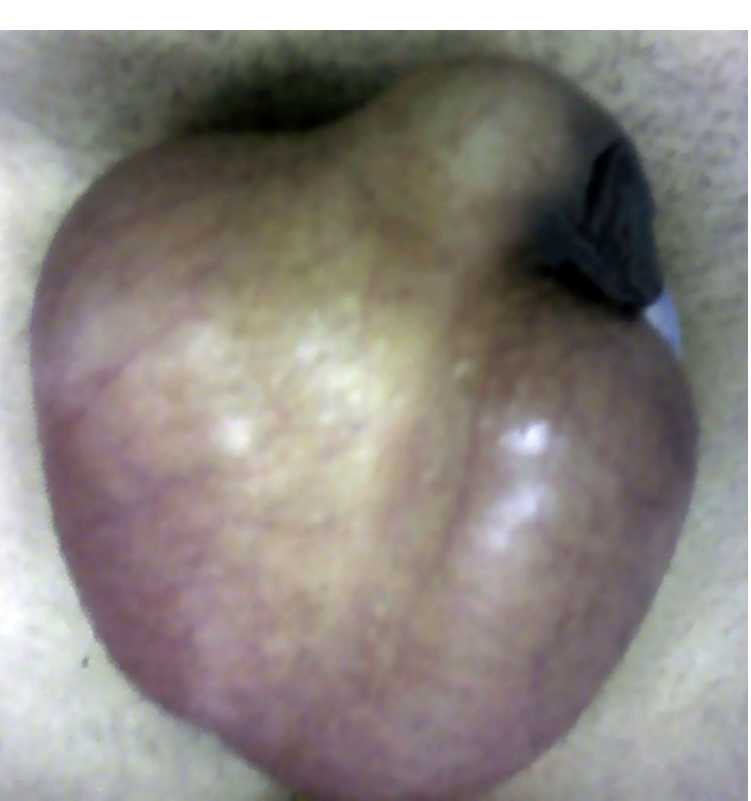
Oedematous scrotum.

**Figure 3:**
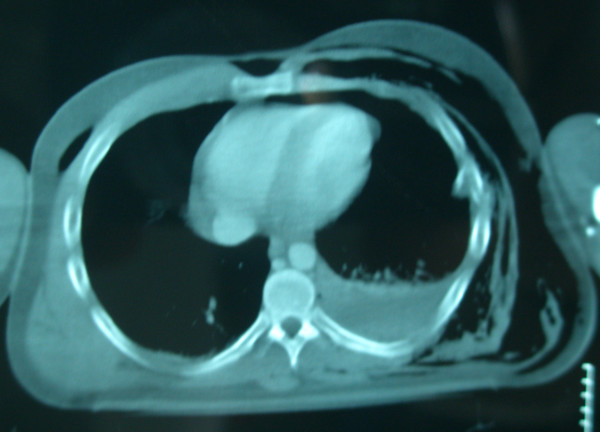
CT demonstrates left pneumo-haemothorax with subcutaneous emphysema.

**Figure 4:**
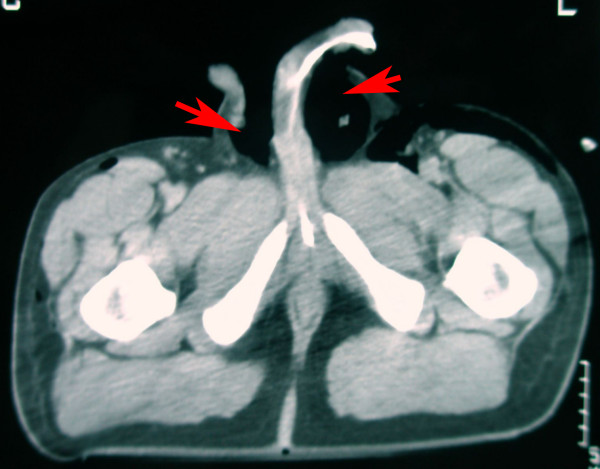
CT demonstrates pneumoscrotum (red arrows).

## Discussion

Subcutaneous emphysema of the scrotum – especially of traumatic etiology – is not a common medical condition. Causes that may result in the presence of air in the scrotum are:

1. Iatrogenic: such as various endoscopies (colonoscopy, polypectomy, laparoscopic procedures, tracheostomy, CPR, chest drain tube insertion, dental procedures) [1-3].

2. Gas producing infection (gangrene Fournier).

3. Pneumothorax [4,5].

4. Scrotal trauma.

5. Visceral perforation [6].

The true nature of the development of the subcutaneous emphysema of the scrotum has not been fully elucidated. Three possible mechanisms were proposed:

1. Rapture of the alveoli and expansion of the air through the mediastinum in the subcutaneous area [6].

2. Expansion of the emphysema along the Scarpa's Fascia [7].

3. Through the mediastinum air passes in the paranephric space and through the retroperitoneum and the inguinal canal is gathered in the scrotum [8-10].

In the case described above, spread of the subcutaneous emphysema towards scrotum is the most "desirable" potential, given the fact that if this expansion was towards neck it could obstruct the upper airways and lead to suffocation.

## Conclusion

We can support that subcutaneous emphysema of the scrotum due to traumatic pneumothorax (blunt trauma), despite its impressive clinical presentation, is not really an urgent situation and assessment should be supportive with intervention directed at the etiology, that is the pneumothorax [11].

## Abbreviations

CT: Computed Tomography; CPR: Cardio Pulmonary Resuscitation.

## Competing interests

The authors declare that they have no competing interests.

## Authors' contributions

All authors have made substantial contribution to concept this case reports

## Consent

Written informed consent was obtained from the patient for publication of this case report and accompanying images. A copy of the written consent is available for review by the Editor-in Chief of this journal.
